# Died

**Published:** 1862-11

**Authors:** 


DIED—At the residence of his father, Dr. Edward Mackay, in this City, on the evening of the 13th ult., Lieut. JAMES C. MACKAY, 63d Regiment N. Y. S. V., aged 23 years.

Lieut. Mackay, with a thorough academic education, had also nearly completed his medical course under the instruction of his father, when he volunteered into the 21st Regiment N. Y. S. V., and served in that capacity until 26th of February, 1862, when he was transferred and promoted to a first Lieutenancy in the 63d N. Y. S. V. He was soon selected by General Meagher as one of his staff, and at the battle of Antietam was struck with a rifle-ball in the hip, while carrying an order from General Meagher to the Colonel of the 63d, and immediately fell from his horse. His father, on learning of his wound, visited him, and again returned with *great difficulty* and effort, bringing his son with him to Buffalo, to die in the quiet of home. His noble qualities of head and heart had won him many friends who will deeply mourn his death. General Meagher speaks of his bravery in terms of highest praise, and also of the regard entertained for him by every officer of the brigade.

